# Advancing Peripheral Nerve Graft Transplantation for Incomplete Spinal Cord Injury Repair

**DOI:** 10.3389/fncel.2022.885245

**Published:** 2022-04-28

**Authors:** Jacob Kjell, Mikael Svensson

**Affiliations:** ^1^Department of Clinical Neuroscience, Karolinska Institutet, Solna, Sweden; ^2^Neurosurgery, Karolinska University Hospital, Solna, Sweden

**Keywords:** spinal cord injury, peripheral nerve graft, axon regeneration, repair strategy, chronic injury, sensorimotor functional recovery

## Abstract

Peripheral nerves have a propensity for axon growth and regeneration that the central nervous system lacks (CNS). However, CNS axons can also grow long distances if introduced to a graft harvested from a peripheral nerve (PNGs), which is the rationale for using PNGs as repair strategy for injuries of the spinal cord. From a clinical perspective, PNGs provide interesting possibilities with potential to repair the injured spinal cord. First, there are numerous options to harvest autologous grafts associated with low risk for the patient. Second, a PNG allow axons to grow considerable distances and can, by the surgical procedure, be navigated to specific target sites in the CNS. Furthermore, a PNG provides all necessary biological substrates for myelination of elongating axons. A PNG can thus be suited to bridge axons long distances across an injury site and restore long tracts in incomplete SCI. Experimentally, locomotor functions have been improved transplanting a PNG after incomplete injury. However, we still know little with regard to the formation of new circuitries and functional outcome in association to when, where, and how grafts are inserted into the injured spinal cord, especially for sensory functions. In this perspective, we discuss the advantages of PNG from a clinical and surgical perspective, the need for adding/repairing long tracts, how PNGs are best applied for incomplete injuries, and the unexplored areas we believe are in need of answers.

## Introduction

Spinal cord injury (SCI) affects millions world-wide and each year there are more than 500 thousand new cases, caused by accidents, violence, and surgical intervention (National Spinal Cord Injury Statistical Center, 2021). In comparison to many other disorders, the functional losses as a consequence of the injury remain permanent and there is no clinically available therapeutic treatment. Most injuries are incomplete, with partial loss of function below the injury. Hence, the loss of function among the majority of patients can be quite heterogeneous, which is further exaggerated by difference in the potential for spontaneous recovery depending on the injury location and the secondary sequalae that hinders repair (Kjell and Olson, 2016). Despite some spontaneous recovery in the first months after an incomplete injury, most of the initially lost functions will remain as chronic deficits (Fawcett et al., 2007; Kirshblum et al., 2021). Development of potential new treatments for chronic injuries are in the minority, while most interventions are aimed at the acute and subacute stage. For clinical translation acute-subacute treatments are very challenging as large groups of patients must be studied due to highly variable outcome in patients with similar acute symptoms. There are also ethical concerns about asking newly injured, often desperate patients, to participate in clinical trials. Hence, there is a need for treatments that can address obstacles and complexities associated to various chronic conditions.

Although the tracts and neurons of the spinal cord have a fair ability for shorter regeneration and plasticity, it is important to keep in mind that functional recovery remains dependent on spared long tracts that maintain adequate conductive properties (Rossignol, 2006; Chew et al., 2012). Therefore, we believe it is fair to hypothesize that a combination of circuit rearrangement and an addition of long tracts would maximize the potential for recovery of functions below the injury site. However, long distance growth within the injured spinal cord is a challenge that seems difficult to overcome (Chew et al., 2012). Given the original spinal circuitry architecture, 10 cm and beyond of robust axonal growth may be needed for directly targeting and providing any recovery of specific functions. One of the few treatment interventions that have shown the potential of providing this type of repair is transplantation of long axon growth-promoting peripheral nerve grafts (PNGs) into the spinal cord (Côté et al., 2011). However, although it has unique potential, several details regarding the new circuitry formation and function, outcome as a result of various surgical placements, and application at a chronic stage remain undetermined. In this perspective, we wish to discuss the need for adding/repairing long tracts and also put it in the context of the last decades progress with regard to our understanding of spontaneous recovery and circuit rearrangements. Also, we will discuss the advantages of PNGs from a clinical and surgical perspective, explain our perspective of the most advantageous application of PNG for incomplete SCIs, and highlight unexplored areas we believe are in need of answers.

### The Obstacle at the Center of It All

The uninjured human spinal cord carries sensorimotor signals back and forth from the periphery to the brain through long and short neuronal connections through the of 43–45 cm length of the spinal cord. Axons and dendrites of the spinal cord typically only regenerate short distances, while the nerves in the periphery can heal great lengths (Scheib and Höke, 2013). In the injured spinal cord, there will be progressive scarring at the injury site that inhibits regeneration and becomes a wall that the spinal circuitry has to circumvent for any spontaneous recovery and regeneration (Norenberg et al., 2004; Silver et al., 2014; Kjell and Olson, 2016). In many cases, if there is edema at the injury site, a cyst may develop instead or as an addition to the scar. The injury site therefore remains a chronic obstacle that will vary in form and shape for each individual that sustained an incomplete injury. Our observations delineate that the injury site in traumatic injuries typically span 1.5–6 cm in length, whether the injury is complete or incomplete (Frostell et al., 2012). Hence, therapeutic transplant has to be able to adjust to various lengths of the injury, while enabling long axonal regeneration or long neuronal relay connections within the spinal tissue. This is a fact that makes injury site-directed surgical interventions complicated. The components of the spinal scar have been extensively reviewed elsewhere and although experimental treatments that are promising for dealing major inhibitory components, regeneration of tracts for longer distances within the spinal cord remain a daunting task (Silver et al., 2014; Bradbury and Burnside, 2019). Instead of attempting to bring long tracts through the scar, taking axons around the injury site and past long stretches of spinal cord tissue would be a way to provide repair of sensorimotor functions below the injury site. In fact, this type of repair occurs after incomplete injuries when spared tracts or long propriospinal circuitry take over some of the functions previously performed by severed tracts during the spontaneous recovery that ensues during the first year after injury (Rossignol, 2006; Fawcett et al., 2007; Kirshblum et al., 2021).

### Spontaneous Recovery and Intact Long Circuitry

In the last two decades, we have made many advances in our understanding of the neurobiology behind spontaneous recovery after SCI. After an incomplete SCI, there are great amounts of new shorter sprouting, plasticity, and compensation both in propriospinal and supraspinal circuitry (Bareyre et al., 2004; Courtine et al., 2008; Flynn et al., 2011; Zörner et al., 2014). Firstly, after incomplete injuries new intraspinal relay circuits provide spontaneous recovery (Bareyre et al., 2004; Courtine et al., 2008). Secondly, spared axons may sprout new collaterals connecting to adjacent circuitry (Hagg, 2006). New connections may also form at various levels supraspinally to adapt to new input (Zörner et al., 2014). Lastly, there is synaptic plasticity that adapts to functional need and new signaling. Overall, this allows signals and functions to relocate to new circuitry setups. Recently, it has been found that locomotor recovery after an incomplete injury is also dependent functional local proprioceptive feedback circuits at various levels below the injury (Takeoka et al., 2014; Takeoka and Arber, 2019). Nevertheless, spontaneous functional recovery after incomplete SCI remains dependent on intact longer tracts that take signals to and from the brain.

Importantly, the spontaneous recovery after SCI is in itself limited, primarily demonstrated by the chronic loss of function by patients with SCI. However, it is possible to enhance process associated to spontaneous recovery and promote further functional repair (Duffell and Donaldson, 2020). The classic way of enhancing functional ability is through, often arduous, rehabilitation programs (Fouad and Tetzlaff, 2012). Lately, using our understanding of these systems and how electric stimulation can activate them, we may in the future reliably push spontaneous recovery further with the circuitry architecture that remains (Wagner et al., 2018). Nevertheless, the ability to enhancing spontaneous recovery also depend on various intact and long circuitry in the spinal cord. To what degree a tract, either extending supraspinally or part of the propriospinal network, can compensate and support functional signaling, and whether there is a ceiling or diminishing return is unknown. However, it has been reported that either spared or new connections comprising about 5–10 percent of the original tract can compensate for large parts of the original function (Hollis et al., 2015). Hence, regenerating or adding even a smaller part of long reaching tracts may potentially provide valuable functional recovery.

### Peripheral Nerve Graft Repair

Experimentally, long circuitry reconstruction after SCI has only been achieved with PNGs transplantation (Côté et al., 2011). This unique position rests on the fact that axons of the central nervous system (CNS) can grow great lengths in a peripheral nerve, very much similar to the repair seen with peripheral axons (Scheib and Höke, 2013). PNG as a method for spinal cord repair and its history has been reviewed in detail by Houle and colleagues and we will focus on describing some key concepts here (Côté et al., 2011). Importantly, the axons in the PNG will be myelinated by the Schwann cells, in contrast to many regenerating axons in the spinal cord that remain unmyelinated or poorly myelinated (Tan et al., 2007; James et al., 2011). The grafts are autologous and do not need additional immunosuppression to avoid graft rejection. Furthermore, surgical placement of a PNG can reduce the distances axons need to travel when entering or exiting the graft. Axons entering and exiting the graft can be increased by improving the microenvironment around the insertion points with various co-treatments, such as adding FGF1 in the tissue glue used during grafting (Cheng et al., 1996; Lee et al., 2008; Nordblom et al., 2012). Therapeutic PNG techniques have primarily been advanced toward clinical application as a treatment intended for complete SCI (Nordblom et al., 2009; Frostell et al., 2018).^1^ In injuries with no function below the injury site, the transplantation involves first completely removing the spinal scar and then reconnecting the disconnected spinal cord with several short PNG assigned in locations that will direct spinal tract regeneration from white matter into the opposing spinal gray matter (Cheng et al., 1996). In the case of incomplete SCIs, the intervention typically involves grafting one end of a peripheral nerve at or close to the injury site and the severed tracts, and the other end close to neuronal targets. This effectively makes the PNG a bridge that may allow spinal cord axons access to distant locations. In this manner, a single PNG transplanted as a “bridge” connecting spinal locations two spinal levels apart has been shown effective in improving either locomotor or respiratory recovery (Houle et al., 2006; Alilain et al., 2011). However, advancement of various other transplant strategies have reached a stage where it is reasonable to believe they may also be able to provide some repair in motor functions two spinal sections below the injury (Rosenzweig et al., 2018). Nevertheless, we would like to argue that PNG still is an interesting repair strategy for incomplete injuries with its unique capability to provide long axon growth-promoting bridges that can be surgically placed at various location on the spinal cord. However, there are still several gaps in our knowledge of using long PNGs “bridges” for repair of various types of incomplete spinal cord injuries.

### Peripheral Nerve Graft for Incomplete Injuries

For incomplete injuries, surgery and PNG transplantation would be performed in an individual with some functions remaining below the injury site. Prior to a surgical proceedure associated to any such injury, a proper assessment of the injury-outcome, the potential benefits, and risks would be necessary. Such an assessment, asserted with great confidence, is only really feasible in the chronic stage. Therefore, we believe implementation of a surgical intervention such as PNG would be most practical and reasonable in the chronic state, when spontaneous recovery has ended and scarring has matured. We reason that any application in humans would depend on minimizeing transplantation-associated injury by positioning one end of the graft at the border of the scar, while the other end would be grafted close to gray matter either sub-pially or longitudinally between white matter tracts. With this method, the scar is bypassed and tissue damage to the spinal cord is minimized. Nevertheless, the small injuries to the spinal cord associated to the transplantation may be considered a draw back and an unavoidable potential risk. Notably, experiments in rodents suggest removing the scar may be possible without functional harm (Sandrow et al., 2008). Although, removing the spinal scar in an incomplete injury would be clinically unfeasible today, it may be considered an acceptable risk in the future if proper techniques are developed that can, with certainty, delineate the spinal scar borders. Lastly, we deem it possible to surgically place the graft itself in the spinal column, above or below the dura, so it remains protected. With regard to the various steps in the surgery method, it could be advantageous to develop the surgical application in large animal models.

From the clinical stand-point, an important advantage of using PNG to bypass the injury site is that the surgical intervention can be adapted depending the injury of the individual. The intervention can be adjusted based on the injury location, results from neurological examination, and the intended area of functional recovery. We have shown in a previous study that the upper and lower level of an injury can be determined with high precision in the thoracic spine (Frostell et al., 2012). In order to extract such information, we used standard neurophysiological evaluation in combination with MRI. MRI alone will not give this information since it doesn’t tell anything about the functional status of the cord. We believe that functional data is valuable when any surgical approach to the injured cord is considered. In particular, when transplantation procedures like cell therapy or corresponding is considered to minimize risk by having precise information about the level of injury mapped before surgical intervention. Technically, we solved this using a bipolar needle for EMG registrations stepping through all intercostal muscles. Voluntarily activated motor units were registered above the injury, thus giving data on the upper level. Normal motor units can also be generated below the injury by inducing spasticity in the in lower limbs in parallel with EMG registration, which allows mapping of the lower border of the injury zone.

**FIGURE 1:**
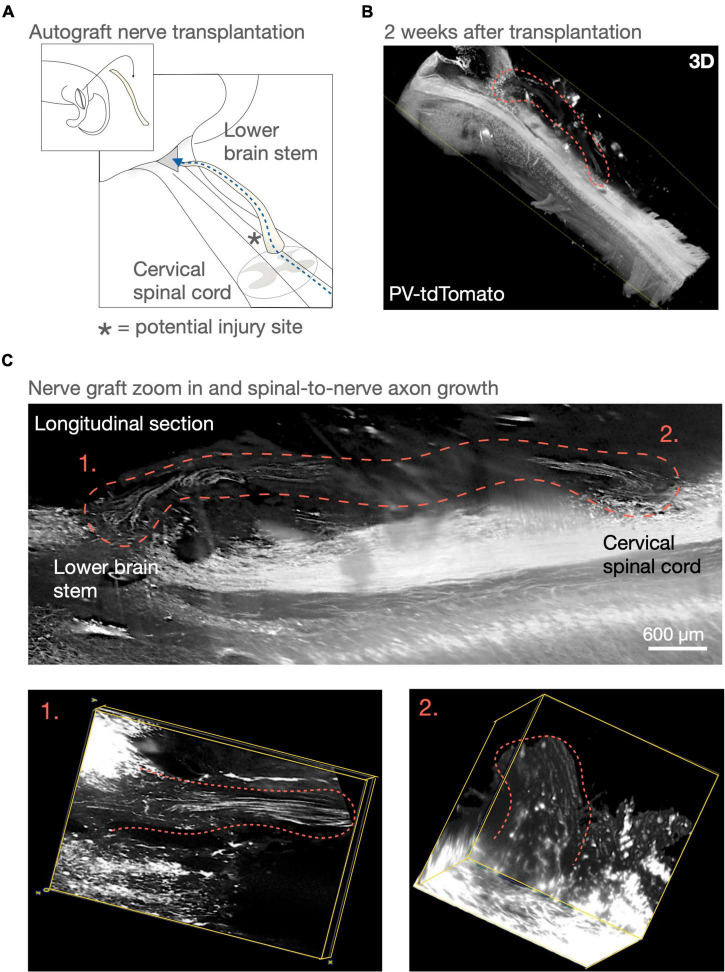
Peripheral nerve graft (PNG) and repairing the sensory tracts of the dorsal column. In 1981, David and Aguayo (1981) demonstrated long distance axon growth using a peripheral nerve as a bridge for dorsal column axons. This experimental model aimed at demonstrating long-distance regeneration. However, using one or more PNGs as an axon bypass in a similar manner could be a potential repair strategy for incomplete spinal cord injuries that wouldn’t necessitate removing the spinal scar. **(A,B)** Here in a similar surgery model to David and Aguayo’s (1981) PNG “bridge” model, we visualize parts of the sensory tracts of the dorsal column in the lower brain stem and cervical spinal cord using a parvalbumin-reporter mouse (PV-tdTomato mouse). **(C)** An autologous PNG is transplanted and 2 weeks after transplantation we can visualize axons throughout the PNG. To follow axons throught the cord in 3D was not possible at the time of Aguayo’s (1985) experiment and our understanding of using PNG as a repair strategy for the spinal cord has advanced. Such techniques and knowledge may today allow us to obtaining necessary answers for potentially considering advancing PNG transplantation as a long-distance axonal tract repair for incomplete spinal cord injuries.

With the clinical application in mind, questions regarding the potential for functional repair would need an experimental model that aims to reconnect long distances and remove/injure less tissue than done before, while primarily performing transplantations in the chronic stages (Houle, 1991; Alilain et al., 2011; Côté et al., 2011; see Figure 1). Among the first PNG experiments in the spinal cord, David and Aguayo (1981) inserted a PNG bridging the sensory tracts of the dorsal column of the cervical spinal cord back up to the cuneate and gracile nuclei of the brains stem (David and Aguayo, 1981; Aguayo, 1985). The PNG allowed, at that time, unprecedented growth from adult CNS axon and it also encouraged the idea that parts of severed spinal tracts perhaps could be restored. Although functional regeneration has been demonstrated for motor function since then, it still remains to be demonstrated that PNG can restore sensory function in an incomplete SCI (Houle et al., 2006). For investigating such a sensory axon bypass, a similar model used by Aguayo et al. could be advantageous (see Figure 1). Notably, the dorsal column lends itself well to various modern imaging and trancing tools (Attwell et al., 2018; Susaki et al., 2019) (Figure 1). Moreover, and clinically incomplete SCI often involve the dorsal columns of the spinal cord. Also, to recover some sensory function below the injury could be valuable, since it may for example allow individual with SCI to prevent pressure ulcers. Furthermore, the clinical experience concerning injuries of the dorsal column involves severe neurological deficits with significant gait disturbance (Nathan, 1994). In rodents, the functional loss is also severe initially. However, with time, rodents recover and compensate for most of the functional loss. Recently, it was discovered that this recovery is due to approximately 6% of the dorsal column tracts that sprout ventrolaterally to intact tracts that are part of the same circuitry network and also reaches the thalamus in the brain (Hollis et al., 2015). Putting PNG in context of this type of spontaneous recovery will be important for its implementation and it will be necessary to understand how and if this affects the propensity for a PNG to provide functional recovery in the context of a chronic injury.

A robust model of PNG transplantation for incomplete injury will allow investigation of other important questions regarding the details of axon growth through the graft and functional connectivity at the nuclei. After Aguayo’s (1985) original experiments, it was found that axons in the graft originated both from axons of the ascending tracts and local propriospinal neurons close to the graft insertion point (David and Aguayo, 1981; Aguayo, 1985). There were both axons running up and down the graft and their identity seemed varied from electrophysiological measurements. Today, new PNG surgery techniques have been developed, for example using different grafting time-points for the caudal and rostral end of the inserted graft (Houle et al., 2006; Alilain et al., 2011; Côté et al., 2011), which can reduce the number of axons in the opposite direction. In the successful experiments with complete SCI, the graft was apposed the white matter and directed onto gray matter (Cheng et al., 1996). Whether precise apposition of PNG onto white matter tract such as the dorsal column and other end directly apposed the gray matter of the cuneate or gracile nuclei will provide more axon growth from primarily axonal tracts, rather than local neurons, is unknown. Furthermore, the neuronal identity of axons entering and exiting the graft, as well as functional connectivity and conduction properties, will need to be studied in detail. Whether graft source makes a difference and in what ways graft length makes a difference also remains to be studied.

Lastly, with the answers to the above questions in one model system, the crucial conceptual question of; “Does circuitry repair using PNG vary for different tract and/or at different levels?” remains. Hence, comparing the outcome of PNGs for some different and clinically relevant incomplete injuries is necessary. It would provide answer to the overall question of; What could surgical PNG treatment mean for functional recovery of incomplete injuries? One thing to consider here is, given what we have learned from spontaneous recovery and introducing new relay neurons, that it may not be necessary to repair tracts in their original form in order to provide robust functional improvements (Courtine et al., 2008; Fischer et al., 2020; Zavvarian et al., 2020). By understanding the potential or need for relay circuitry for PNG better, the grafts technique may be found useful in unexpected ways. Perhaps, spinal locations distant to the injury site can be reconnected by simply reaching other tracts able to pass the injury site. Or perhaps PNG in combination with growth prone-relay circuits derived from transplanted neural stem cell can provide better outcome, especially in a chronic SCI (Rosenzweig et al., 2018; Fischer et al., 2020). Furthermore, combining PNG with exercise already proven beneficial (Houle and Côté, 2013). Hence, site-directed electrical stimulation may be a way to boost both axon growth and new circuitry formation with PNGs (Jack et al., 2020).

## Conclusion

Peripheral nerve graft as an experimental therapeutic treatment for SCI has demonstrated that it can provide improved recovery even for incomplete injuries. Transplantation of PNGs has a unique potential to bypass damaged long tracts, providing myelination, and be surgically directed toward specific sites of the spinal cord. Hence, it could potentially provide a functional recovery for patients simply not possible with other experimental treatments. However, we believe that this implementation and its advancement toward clinical application hinges on addressing some conceptual questions and developing of appropriate surgery. Nevertheless, given that the above discussed knowledge gaps are addressed in the future we believe that a very large group of individuals would benefit from the type of repair that PNG implants may offer.

## Data Availability Statement

The original contributions presented in the study are included in the article/supplementary material, further inquiries can be directed to the corresponding authors.

## Ethics Statement

The animal study was reviewed and approved by the regional Animal Ethics Committee in Stockholm.

## Author Contributions

JK and MS wrote the perspective. MS conceived the project and performed surgery. JK conceptualized and planned the project and performed tissue analysis. Both authors contributed to the article and approved the submitted version.

## Conflict of Interest

The authors declare that the research was conducted in the absence of any commercial or financial relationships that could be construed as a potential conflict of interest.

## Publisher’s Note

All claims expressed in this article are solely those of the authors and do not necessarily represent those of their affiliated organizations, or those of the publisher, the editors and the reviewers. Any product that may be evaluated in this article, or claim that may be made by its manufacturer, is not guaranteed or endorsed by the publisher.

## Footnotes

1
https://clinicaltrials.gov/ct2/show/NCT02490501
